# Grief symptoms and primary care use: a prospective study of family caregivers

**DOI:** 10.3399/bjgpopen20X101063

**Published:** 2020-06-10

**Authors:** Mette Kjærgaard Nielsen, Kaj Christensen, Mette Asbjorn Neergaard, Pernille Envold Bidstrup, Mai-Britt Guldin

**Affiliations:** 1 Research Unit for General Practice, Aarhus, Denmark; 2 Department of Public Health, Aarhus University, Aarhus, Denmark; 3 Department of Oncology — The Palliative Care Unit, Aarhus University Hospital, Aarhus, Denmark; 4 Psychological Aspects of Cancer, Danish Cancer Society, Copenhagen, Denmark; 5 Department of Psychology, University of Copenhagen, Copenhagen, Denmark

**Keywords:** bereavement, grief, caregivers, psychology, psychotropic drugs, primary health care

## Abstract

**Background:**

Family caregivers to patients who are severely ill have high use of primary health care and psychotropic medication. However, it remains sparsely investigated whether healthcare services target the most vulnerable caregivers.

**Aim:**

This study aimed to examine associations between family caregivers’ grief trajectories of persistent high-grief symptom level (high-grief trajectory) versus persistent low-grief symptom level (low-grief trajectory), as well as early contacts with GPs or psychologists and the use of psychotropic medication.

**Design & setting:**

A population-based cohort study of family caregivers (*n* = 1735) in Denmark was undertaken.

**Method:**

The Prolonged Grief-13 (PG-13) scale measured family caregivers’ grief symptoms at inclusion (during the patient's terminal illness), 6 months after bereavement, and 3 years after bereavement. Multinomial regression was used to analyse register-based information on GP consultations, psychologist sessions, and psychotropic medication prescriptions in the 6 months before inclusion.

**Results:**

A total of 1447 (83.4%) family caregivers contacted their GP, and 91.6% of participants in the high-grief trajectory had GP contact. Compared with family caregivers in the low-grief trajectory, family caregivers in the high-grief trajectory had ≥4 face-to-face GP consultations (odds ratio [OR] = 2.6; 95% confidence interval [CI] = 1.3 to 5.0), more GP talk therapy (OR =4.4; 95% CI = 1.9 to 10.0), and more psychotropic medication, but not significantly more psychologist sessions (OR = 1.7; 95% CI = 0.5 to 6.6).

**Conclusion:**

Family caregivers in the high-grief trajectory had more contact with their GP, but their persisting grief symptoms suggest that primary care interventions for family caregivers should be optimised. Future research is warranted in such interventions and in the referral patterns to specialised mental health care.

## How this fits in

Family caregivers have high use of primary care services before and after bereavement. Persistent grief symptoms indicate that family caregivers need support from health professionals. This study shows a higher use of primary care services and psychotropic medication before bereavement in family caregivers who developed persistent high-grief symptoms. Early identification of vulnerable family caregivers and development of targeted interventions adapted to primary care is indicated.

## Introduction

Severe illness may cause grief and distress in patients and their relatives.^1^ A substantial proportion of family caregivers report high levels of grief symptoms (15%),^2,3^ caregiver burden (11%–33%),^4,5^ and anxiety and depressive symptoms (15%–30%).^1,5^ Previous studies have shown that family caregivers are prescribed more antidepressants and sedatives during end-of-life care and bereavement,^6,7^ have more psychologist sessions before and after bereavement,^6^ are hospitalised more often,^6,8^ and have higher all-cause mortality^9^ than non-bereaved family caregivers. These prior findings underline the highly stressful situation of most family caregivers before and after bereavement, and call for the identification of support needs at an early time point.

A high level of grief symptoms before bereavement has been found to predict negative reactions after bereavement.^3,10,11^ Prior psychiatric illness, including depressive symptoms, have repeatedly been associated with severe bereavement outcomes such as self-harm, suicide,^12^ depression,^11,13^ and prolonged grief disorder.^11,13,14^ In a previous study of longitudinal development of grief symptoms from end-of-life care into 3 years of bereavement, it was found that 38% of a population-based cohort of family caregivers (*n* = 1735) had persistently low levels of grief symptoms (low-grief trajectory).^15^ Approximately half of the family caregivers had moderate or high grief symptom levels before bereavement, which decreased after death; moderate/decreasing grief trajectory (29%) and high/decreasing grief trajectory (18%). Moreover, 9% had relatively low-grief symptom levels before the patient’s death, which increased at 6 months after the patient’s death (late-grief trajectory), and 6% had persistently high-grief symptom levels (high-grief trajectory). Hence, a substantial group of family caregivers seem to experience grief symptom levels that could suggest unmet needs for support from health professionals such as their GP, psychologist, or psychiatrist.

Continuity of care has been found to reduce patients’ mortality,^16^ and GPs have an opportunity to provide continuous support before and after bereavement. However, it is not known whether the available healthcare services reach the family caregivers who display persistent and severe grief reactions.

This study aimed to investigate associations between grief trajectories in family caregivers and the number of different types of GP consultations, GP-referred psychologist sessions, and use of psychotropic medication (antidepressives and sedatives) before bereavement.

## Method

### ​Study design and setting

This prospective study is based on a longitudinal, population-based cohort of family caregivers in Denmark, where health care is tax-funded and services are free of charge for citizens. The GPs are paid on a per-capita basis combined with fee-for-service payment, and serve as gatekeepers to secondary care.^17^ Psychiatric services are fully covered by the healthcare system. Yet, 40% of the costs of up to 12 GP-referred psychologist sessions are self-paid, whereas up to seven GP talk therapy sessions are free of charge.^18^

**Figure 1.**
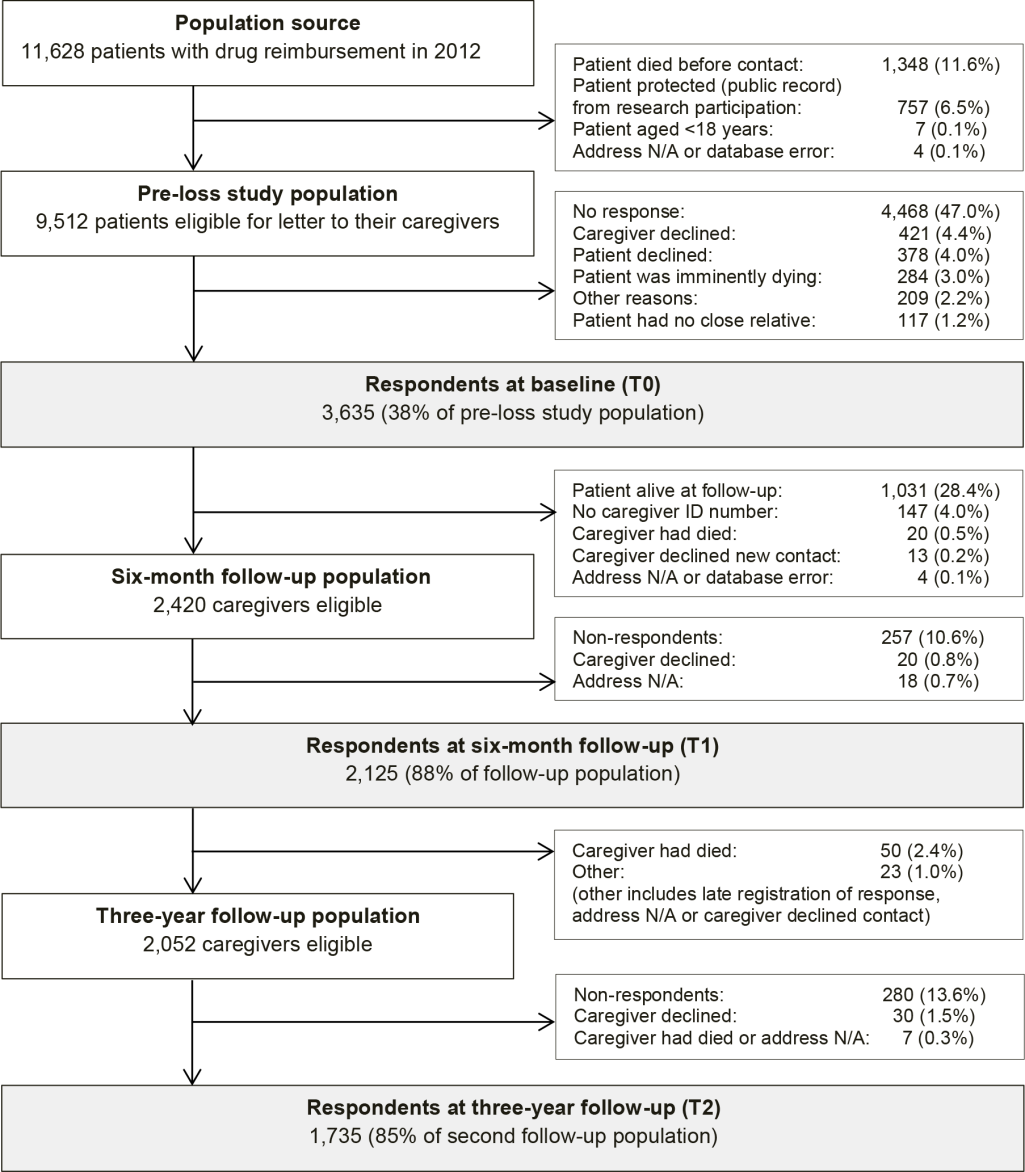
Flow diagram of family caregivers at the time of the patient’s drug reimbursement registration owing to terminal illness (T0), 6 months after the patient’s death (T1), and 3 years after the patient’s death (T2).

To systematically identify family caregivers of patients with terminal illness, register-based information was obtained on all patients receiving drug reimbursement owing to terminal illness in 2012.^19^ On a weekly basis, a letter was sent with a questionnaire to newly registered patients and asked for their closest family caregiver to complete the questionnaire.^5^ Enrolled family caregivers completed a questionnaire at the time of inclusion (T0), at 6 months after bereavement (T1), and at 3 years after bereavement (T2) (Figure 1). It was intended that follow-up at 6 months post-loss would capture short-term grief reactions beyond acute grief, and that 3 years of follow-up would capture long-term grief reactions. Questionnaire data were combined with register-based data variables and analysed at Statistics Denmark.^20^

### ​Questionnaire-based variables

The PG-13 scale^21^ measured grief symptoms at T0, T1, and T2. At baseline (T0), an adapted version of the scale^5^ was used to accommodate to the situation before the patient’s death in line with prior studies.^3^ At baseline, information on the family caregiver’s personal relation to the patient who was terminally ill (partner, non-partner [adult child, other relation]) was obtained.

### ​Register-based variables

Data on the family caregiver’s age, sex, educational level (low [≤10 years], intermediate [>10 and ≤15 years], high [>15 years]), and the patient’s survival time from drug reimbursement initiation were extracted from the Danish Civil Registration System.^22^ The family caregiver’s potential somatic diseases registered in the Danish National Patient Registry^23^ on the basis of hospital contacts were categorised (0, ≥1) according to the Charlson Comorbidity Index (CCI).^24^ Contacts to the GP and a psychologist (after referral from a GP) restricted to a period of 6 months prior to study inclusion were retrieved from the Danish National Health Service Register.^25^ GP contact was presented as overall variables (any consultation) divided into consultation type (daytime, prevention, talk therapy, phone, email, and home visit). The Danish National Prescription Registry^26^ provided Anatomical Therapeutic Chemical (ATC) Classification System codes for any use of sedatives (MN05C) and antidepressants (MN06A).

### ​Statistical analyses

In a prior study, five specific grief trajectories were identified based on grief symptoms on the PG-13 scale at T0, T1, and T2.^15^ Stata Plugin traj was used for estimating group-based trajectory models^27^ in Stata (version 14).^28^ Fit indices and diagnostic model performance were published as Supplemental Material B.^15^

The distribution of healthcare use was described as proportions in the five grief trajectories. Associations were identified between grief trajectories and use of health care and medication using a multinomial logistic regression model, which was adjusted for age, sex, personal relation, education, somatic illness in the family caregiver, and the time from inclusion to the patient’s death. The outcomes were reported as ORs with 95% CIs.

## Results

### ​Study population

At T0, 3635 family caregivers completed the baseline questionnaire. Of these, 2420 (66.6%) had been bereaved within 6 months after baseline and were sent the first follow-up questionnaire at T1. In total, 2125 (58.5% of the baseline population) participated at T1, and 1735 (47.7%) family caregivers also completed the second follow-up questionnaire at T2, comprising the study population (Figure 1). Participants were predominantly females (70.7%) and partners (65.6%) with a mean age of 62 years (Table 1). The high-grief trajectory was associated with older age and being a partner compared with the low-grief trajectory, whereas no sex difference was present.^15^

**Table 1.** Characteristics of the total study cohort at inclusion (baseline), *N* = 1735.

**Caregiver characteristics**	**Mean (** **95%** **CI** **)**	***n*** **(%)**
Age, years^a^	62.0 (61.5 to 62.6)	
Sex		
Male		508 (29.3)
Female		1227 (70.7)
Personal relation		
Partner		1138 (65.6)
Adult child		476 (27.4)
Other		121 (7.0)
Education		
Low (≤10 years)		449 (25.9)
Intermediate (>10 and ≤15 years)		828 (47.7)
High (>15 years)		458 (26.4)
**Health indicators, diagnoses within 5 years prior to baseline**		
Somatic conditions (CCI^b^)		
0		1457 (84.0)
1		239 (13.8)
≥2		39 (2.2)
Psychiatric diagnosis^c^	
Affective disorder		10 (0.6)
Anxiety or adjustment disorder		12 (0.7)
Other^d^		16 (0.9)
No hospital-based psychiatric diagnosis		1697 (97.8)

a Caregiver’s age at baseline.

b CCI = Charlson Comorbidity Index^24^ (scores recorded in the Danish National Health Service Register).^25^

c Diagnosis in the Danish Psychiatric Centre Research Register within 5 years prior to the time of patient’s medical drug reimbursement.^29^

d Includes schizophrenia, psychotic disorders, personality disorders, obsessive-compulsive disorders, and organic mental disorders.

### ​Use of healthcare services and psychotropic medications

Prior to study inclusion, 83.1% of all family caregivers and 91.6% of family caregivers in the high-grief trajectory had a GP consultation. Hereof, 73.0% had a daytime consultation and 4.3% received talk therapy. Additionally, 2.8% had contact with a psychologist after GP referral, 8.9% were prescribed sedatives, and 9.2% were prescribed antidepressants (Table 2). The number of overall consultations and use of sedatives increased by rising age, whereas younger persons had more psychologist sessions (data not shown).

**Table 2.** Healthcare use in the 6 months before baseline among family caregivers, according to grief trajectory.

	**Total**	**Low grief **trajectory****	**Late grief **trajectory****	**Moderate/decreasing grief trajectory**	**High/decreasing grief trajectory**	**High grief **trajectory****
***N*** **(%)**	1735 (100)	670 (38.6)	122 (7.0)	526 (30.3)	310 (17.9)	107 (6.2)
**Use of GP healthcare services 6 months prior to baseline**						
Any consultation, total^a^						
0	293 (16.9)	144 (21.5)	22 (18.0)	79 (15.0)	39 (12.6)	9 (8.4)
≥1^b^	1442 (83.1)	526 (78.5)	100 (82.0)	447 (85.0)	271 (87.4)	98 (91.6)
Daytime consultations, *n*						
0	468 (27.0)	210 (31.3)	26 (21.3)	142 (27.0)	70 (22.6)	20 (18.7)
1–3	869 (50.1)	333 (49.7)	65 (53.3)	274 (52.1)	152 (49.0)	45 (42.1)
≥4	398 (22.9)	127 (19.0)	31 (25.4)	110 (20.9)	88 (28.4)	42 (39.3)
**Mental healthcare interventions**						
GP talk therapy^a^						
0	1660 (95.7)	653 (97.5)	117 (95.9)	505 (96.0)	291 (93.9)	94 (87.9)
≥1	75 (4.3)	17 (2.5)	5 (4.1)	21 (4.0)	19 (6.1)	13 (12.1)
Psychologist session after GP referral^a^						
0	1687 (97.2)	654 (97.6)	118 (96.7)	512 (97.3)	299 (96.5)	104 (97.2)
≥1	48 (2.8)	16 (2.4)	4 (3.3)	14 (2.7)	11 (3.5)	3 (2.8)
Psychotropic medications^c^						
0 sedatives	1581 (91.1)	632 (94.3)	111 (91.0)	480 (91.3)	276 (89.0)	81 (76.4)
≥1 sedatives	154 (8.9)	38 (5.7)	11 (9.0)	46 (8.7)	34 (11.0)	25 (23.4)
0 antidepressants	1575 (90.8)	623 (93.0)	107 (87.7)	489 (93.0)	268 (86.5)	88 (82.2)
≥1 antidepressants	160 (9.2)	47 (7.0)	15 (12.3)	37 (7.0)	42 (13.5)	19 (17.8)

a Registration of contacts in the Danish National Health Service Register.^25^

b GP consultation includes daytime consultations, chronic care consultations, talk therapy, phone consultations, email consultations, and home visits.

c Registration of redeemed prescriptions in the Danish Register of Medicinal Product Statistics.^26^

**Figure 2.**
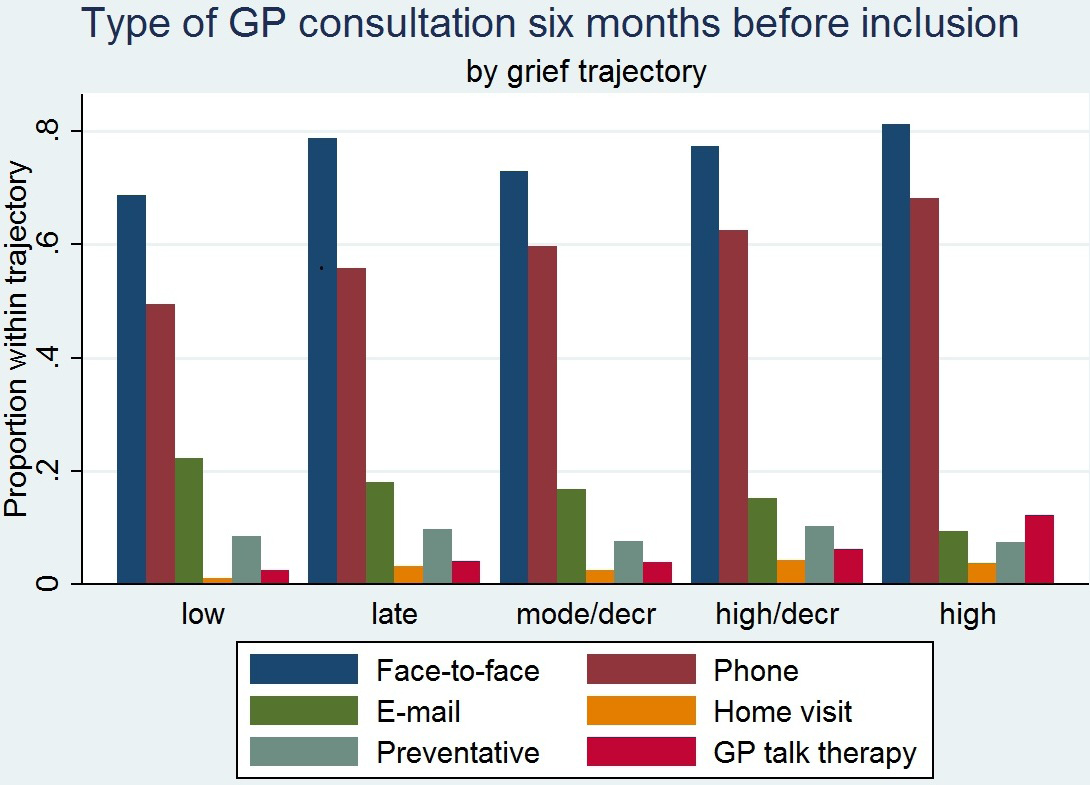
Type of GP consultation 6 months before inclusion. Mode/decr = moderate/decreasing. High/decr = high/decreasing.

Compared with the low-grief trajectory, the high-grief trajectory was associated with ≥4 face-to-face GP consultations (OR = 2.6; 95% CI = 1.3 to 5.0), GP talk therapy (OR = 4.4; 95% CI = 1.9 to 10.0), and the use of sedatives (OR = 3.3; 95% CI = 1.8 to 6.0) and antidepressants (OR = 2.6; 95% CI = 1.4 to 4.7), whereas the number of psychologist sessions did not significantly increase (OR = 1.7; 95% CI = 0.5–6.6) (Table 3). Caregivers in the high-grief trajectory also had more GP phone consultations, but they had fewer email consultations and preventive consultations (Figure 2).

**Table 3.** Consultations and prescriptions during 6 months before inclusion and associations^a^ with grief trajectories, *N* = 1735.

	**Grief trajectory (reference: low grief**)
LateOR (95% CI)	Moderate/decreasingOR (95% CI)	High/decreasingOR (95% CI)	HighOR (95% CI)
GP consultation, any^b^	1.4 (0.8 to 2.6)	1.3 (0.9 to 1.8)	1.6 (1.0 to 2.5)	2.1 (1.0 to 4.5)
GP consultations, face-to-face^c^				
0	1	1	1	1
1–3	2.0 (1.1 to 3.5)	1.0 (0.8 to 1.4)	1.2 (0.8 to 1.7)	1.3 (0.7 to 2.5)
≥4	2.0 (1.0 to 3.8)	1.0 (0.7 to 1.4)	1.5 (1.0 to 2.2)	2.6 (1.3 to 5.0)
GP talk therapy^c^				
0	1	1	1	1
≥1	1.5 (0.5 to 4.3)	1.3 (0.6 to 2.6)	1.7 (0.8 to 3.6)	4.4 (1.9 to 10.0)
Psychologist sessions^c^				
0	1	1	1	1
≥1	2.0 (0.6 to 6.5)	1.1 (0.5 to 2.4)	1.6 (0.7 to 3.9)	1.7 (0.5 to 6.6)
Prescribed medications^d^				
0 sedatives	1	1	1	1
≥1 sedatives	1.2 (0.5 to 2.5)	1.2 (0.7 to 1.9)	1.4 (0.9 to 2.4)	3.3 (1.8 to 6.0)
0 antidepressants	1	1	1	1
≥1 antidepressants	1.8 (0.9 to 3.5)	0.9 (0.6 to 1.5)	1.8 (1.1 to 2.9)	2.6 (1.4 to 4.7)

^a^Any GP consultation includes face-to-face consultations, preventive consultations, GP talk therapy, phone consultations, email consultations, and home visits. ^b^Registration of contacts in the Danish National Health Service Register.^25^ ^c^Registration of redeemed prescriptions in the Danish Register of Medicinal Product Statistics.^26^ ^d^Multinomial regression model adjusted for age, sex, personal relation to the patient, education, somatic illness in the family caregivers (Charlson Comorbidity Index^24^), and time from inclusion to the patient’s death.

## Discussion

### ​Summary

The present study provides new knowledge establishing that family caregivers who developed a high-grief trajectory attended their GP more frequently early in the caregiving period than their comparable peers in a low-grief trajectory; they received more face-to-face consultations and more GP talk therapy, and they were also more likely to be prescribed psychotropic drugs.

During the 6 months prior to the registration of terminal illness, the GP was in contact with the vast majority (83.1%) of family caregivers and with 91.6% of those in a high-grief trajectory. However, 8.4% of this vulnerable group had no contact with their GP. The high-grief trajectory was associated with more face-to-face GP consultations, GP talk therapy, and use of psychotropic medication compared with the low-grief trajectory, whereas the use of a psychologist after GP referral did not differ between grief trajectory groups.

### ​Strengths and limitations

The high completeness of the Danish health registers enabled systematic sampling of a population-based cohort of family caregivers to patients who were severely ill. The considerable sample size of 1735 participants included in the present study enhanced the precision of the estimates and the random sampling improved internal validity. Another strength was the identification of grief trajectories through repeated measurements of grief symptoms on the validated PG-13 scale in the data-driven, group-based trajectory model (GBTM),^27^ which provided an overview of grief development patterns that goes beyond the mean of single measurements of grief symptoms.^15^ A total of 90% completed the PG-13 scale at all time points of the assessment (T0, T1, and T2). Missing values were evenly distributed (missing at random) and handled in the GBTM.

The high-grief trajectory consisted of only 107 individuals, and this small sample size might have been a limitation. Nevertheless, the sample size was larger than in comparable studies and sufficient to adjust for factors considered to be of a priori relevance in the regression analysis,^15^ and further allowed for inclusion of comorbidity in the present analysis of primary care contacts.

The response rate of 38.2% at baseline is comparable with the response rate of family caregivers to severely ill patients in previous studies.^3^ Still, selection bias cannot be ruled out. In a non-response baseline analysis, it was found that patients with study participants were younger and higher educated compared with patients without participants.^5^ When comparing family caregivers at baseline with those included in the present analysis, it was found that the present study population was older and higher educated.^15^ Younger people and those with low education may have been more distressed.^30^ Hence, the use of healthcare services and medication (and the prevalence of adverse grief trajectories) may have been underestimated in this study.

### ​Comparison with existing literature

The findings extend the knowledge gained in prior studies showing overall increased primary healthcare use among family caregivers before bereavement.^6,7,31^ The symptom level in the high-grief trajectory indicates psychological distress in family caregivers and need of support.^15^ Contacts with primary care provides an opportunity for the GP to identify individual support needs and to arrange follow-up visits. A total of 8.4% of family caregivers in the high-grief trajectory had no contact with primary care in the study period. They may have received unregistered support from health professionals, such as community nurses, specialised palliative care units, or self-payed psychologists, without effect on their long-term high-grief symptom levels. Still, it is also possible that they may not have been able to reach out for support, which has previously been reported among 10% of patients with depressive symptoms who did not seek mental health services.^32^ One previous study found that bereaved people were more likely to seek mental health care during bereavement if they had discussed psychological issues with a health professional before bereavement.^33^ An invitation to a designated appointment from health professionals was regarded as positive and helpful among family caregivers in a qualitative study, regardless of the family caregivers’ need for support.^34^ Further research is needed to explore if such a proactive approach by the GP combined with systematic assessment of support needs could ensure that all family caregivers receive the necessary support from the GP and other health professionals.

Although other studies have reported benefits of primary care services during bereavement, the present study findings point at insufficient support for family caregivers with persistently high-grief intensity. Compared with the low-grief trajectory, family caregivers in the high-grief trajectory used more sedatives (23.4% versus 5.7%) and antidepressants (17.8% versus 7.0%). Grief and depression are conditions with clear distinctions although they are connected.^35^ The high use of antidepressants may partly be explained by a concomitant depression, whereas the high use of sedatives prior to the registration of the patient’s terminal illness indicates severe psychological distress. The medical and psychotherapeutic treatment for grief and depression differ, and assessment of symptoms aid provision of targeted support and might prevent unnecessary use of medication.

For bereaved people with persistent high-grief symptom levels, complicated grief therapy has been shown in other studies to significantly alleviate grief reactions, whereas antidepressants have shown effect only on depressive symptoms.^36^ Furthermore, a previous bereavement study showed that GP talk therapy in the period shortly after bereavement reduced the risk of long-term mental illness.^37^ In the present study, the high-grief trajectory was associated with GP talk therapy, which was expected to improve symptom levels. However, grief symptom levels remained high. One reason could be the loosely defined content of GP talk therapy, with no theoretical framework, the format of brief sessions (approximately 20–30 minutes), and especially the limited training of GPs in providing talk therapy.^18^ Also, the situation of family caregivers is complex and is likely to involve allostatic overload.^38^ Caregiving is often a continuous stressor that causes biological dysregulation and overstimulation in those with poor adaptation skills and poses a risk of developing psychiatric illness.^38^ Hence, continuous focus on adaptation skills may be required.

Other resources of mental health support in the Danish healthcare system are psychologist services. In the present study, the caregivers in the high-grief trajectory did not attend psychologists after referral by GPs more than the caregivers in the low-grief trajectory. This might be owing to the timing of assessment (that is, before the registration of the patient’s terminal illness), at which time the need for referral might not yet have been identified. Moreover, waiting time to see a psychologist can be long (up to 1 year) and this may also play a role. Another reason could be that the great demands of caring for a loved one at the end of life may leave little time and surplus energy in the caregiver for therapy. Since psychologist sessions are partly self-paid,^39^ poor economic resources is another likely barrier to help-seeking in those with low socioeconomic positions.^15^ A recent health-check study found that people with low educational level had higher distress levels and that equal opportunity for mental health care is required to ensure optimal support for vulnerable groups.^39^

### ​Implications for practice and research

The vast majority of family caregivers were in contact with their GP before bereavement. This provided a golden opportunity for the GP to identify support needs and plan bereavement care. Health professionals in primary care should consider the need of support in family caregivers and the risk of adverse outcome, as early supportive interventions for vulnerable family caregivers who develop a high-grief trajectory may alleviate symptoms and prevent long-term complications.

A newly developed risk assessment tool has been shown to be feasible in a specialised palliative care setting,^40^ and the Brief Grief Questionnaire can be used to identify grief symptoms in patients in primary health care attending behavioural health services.^41^ Future research needs to examine the feasibility in primary care of a brief risk and symptom identification tool for family caregivers combined with the provision of support; for example, based on evidence from established interventions such as complicated grief therapy.^42^ Intervention components may include psycho-education, network activation, focus on self-care, and meaningful activities. Also, education of health professionals, including GPs, is crucial.^43^ Implementation of interventions may have the potential to support the daily functioning, target the use of psychotropic medication, and prevent complications after bereavement for family caregivers.

## References

[1] Romito F, Goldzweig G, Cormio C (2013). Informal caregiving for cancer patients. Cancer.

[2] Nielsen MK, Neergaard MA, Jensen AB (2017). Preloss grief in family caregivers during end-of-life cancer care: a nationwide population-based cohort study. Psychooncology.

[3] Thomas K, Hudson P, Trauer T (2014). Risk factors for developing prolonged grief during bereavement in family carers of cancer patients in palliative care: a longitudinal study. J Pain Symptom Manage.

[4] Adelman RD, Tmanova LL, Delgado D (2014). Caregiver burden: a clinical review. JAMA.

[5] Nielsen MK, Neergaard MA, Jensen AB (2016). Psychological distress, health, and socio-economic factors in caregivers of terminally ill patients: a nationwide population-based cohort study. Support Care Cancer.

[6] Guldin M-B, Jensen AB, Zachariae R, Vedsted P (2013). Healthcare utilization of bereaved relatives of patients who died from cancer. A national population-based study. Psychooncology.

[7] King M, Vasanthan M, Petersen I (2013). Mortality and medical care after bereavement: a general practice cohort study. PLoS One.

[8] Miles TP, Allegra JC, Ezeamama A (2016). In a longevity Society, loss and grief are emerging risk factors for health care use: findings from the health and retirement survey cohort aged 50 to 70 years. Am J Hosp Palliat Care.

[9] Möller J, Björkenstam E, Ljung R, Yngwe MA (2011). Widowhood and the risk of psychiatric care, psychotropic medication and all-cause mortality: a cohort study of 658,022 elderly people in Sweden. Aging Ment Health.

[10] Nielsen MK, Neergaard MA, Jensen AB (2016). Do we need to change our understanding of anticipatory grief in caregivers? A systematic review of caregiver studies during end-of-life caregiving and bereavement. Clin Psychol Rev.

[11] Nielsen MK, Neergaard MA, Jensen AB (2017). Predictors of complicated grief and depression in bereaved caregivers: a nationwide prospective cohort study. J Pain Symptom Manage.

[12] Guldin M-B, Ina Siegismund Kjaersgaard M, Fenger-Grøn M (2017). Risk of suicide, deliberate self-harm and psychiatric illness after the loss of a close relative: a nationwide cohort study. World Psychiatry.

[13] Stroebe M, Schut H, Stroebe W (2007). Health outcomes of bereavement. Lancet.

[14] Shear MK (2015). Clinical practice. complicated grief. N Engl J Med.

[15] Nielsen MK, Carlsen AH, Neergaard MA (2019). Looking beyond the mean in grief trajectories: a prospective, population-based cohort study. Soc Sci Med.

[16] Pereira Gray DJ, Sidaway-Lee K, White E (2018). Continuity of care with Doctors-a matter of life and death? A systematic review of continuity of care and mortality. BMJ Open.

[17] Pedersen KM, Andersen JS, Søndergaard J (2012). General practice and primary health care in Denmark. J Am Board Fam Med.

[18] Davidsen AS (2010). [Talking therapy as part of the general practitioner’s normal working day]. [Article in Danish]. Ugeskr Laeger.

[19] Johannesdottir SA, Horváth-Puhó E, Ehrenstein V (2012). Existing data sources for clinical epidemiology: the Danish national database of Reimbursed prescriptions. Clin Epidemiol.

[20] Schmidt M, Schmidt SAJ, Adelborg K (2019). The Danish health care system and epidemiological research: from health care contacts to database records. Clin Epidemiol.

[21] Prigerson HG, Horowitz MJ, Jacobs SC (2009). Prolonged grief disorder: psychometric validation of criteria proposed for DSM-V and ICD-11. PLoS Med.

[22] Schmidt M, Pedersen L, Sørensen HT (2014). The Danish civil registration system as a tool in epidemiology. Eur J Epidemiol.

[23] Schmidt M, Schmidt SAJ, Sandegaard JL (2015). The Danish national patient registry: a review of content, data quality, and research potential. Clin Epidemiol.

[24] Thygesen SK, Christiansen CF, Christensen S (2011). The predictive value of ICD-10 diagnostic coding used to assess Charlson comorbidity index conditions in the population-based Danish national Registry of patients. BMC Med Res Methodol.

[25] Andersen JS, Olivarius NDF, Krasnik A (2011). The Danish National health service register. Scand J Public Health.

[26] Kildemoes HW, Sørensen HT, Hallas J (2011). The Danish national prescription registry. Scand J Public Health.

[27] Nagin DS, Odgers CL (2010). Group-Based trajectory modeling in clinical research. Annu Rev Clin Psychol.

[28] Jones BL, Nagin DS (2012). A Stata plugin for estimating group-based trajectory models. https://ssrc.indiana.edu/doc/wimdocs/2013-03-29_nagin_trajectory_stata-plugin-info.pdf.

[29] Mors O, Perto GP, Mortensen PB (2011). The Danish psychiatric central research register. Scand J Public Health.

[30] Geyti C, Maindal HT, Dalsgaard EM (2017). Mental health assessment in health checks of participants aged 30–49 years: a large-scale cohort study. Prev Med Reports.

[31] Oksuzyan A, Jacobsen R, Glaser K (2011). Sex differences in medication and primary healthcare use before and after spousal bereavement at older ages in Denmark: nationwide register study of over 6000 bereavements. J Aging Res.

[32] Packness A, Halling A, Hastrup LH (2018). Socioeconomic position, symptoms of depression and subsequent mental healthcare treatment: a Danish register-based 6-month follow-up study on a population survey. BMJ Open.

[33] Lichtenthal WG, Nilsson M, Kissane DW (2011). Underutilization of mental health services among bereaved caregivers with prolonged grief disorder. Psychiatr Serv.

[34] Adams E, Boulton M, Rose PW (2012). A qualitative study exploring the experience of the partners of cancer survivors and their views on the role of primary care. Support Care Cancer.

[35] Bonanno GA, Neria Y, Mancini A (2007). Is there more to complicated grief than depression and posttraumatic stress disorder? A test of incremental validity. J Abnorm Psychol.

[36] Shear MK, Reynolds CF, Simon NM (2016). Optimizing treatment of complicated grief: a randomized clinical trial. JAMA Psychiatry.

[37] Fenger-Grøn M, Kjaersgaard MIS, Parner ET (2018). Early treatment with talk therapy or antidepressants in severely bereaved people and risk of suicidal behavior and psychiatric illness: an instrumental variable analysis. Clin Epidemiol.

[38] McEwen BS (1998). Stress, adaptation, and disease. allostasis and allostatic load. Ann N Y Acad Sci.

[39] Geyti C, Dalsgaard E-M, Sandbæk A (2018). Initiation and cessation of mental healthcare after mental health screening in primary care: a prospective cohort study. BMC Fam Pract.

[40] Thomsen KT, Guldin M-B, Nielsen MK (2017). A process evaluation of systematic risk and needs assessment for caregivers in specialised palliative care. BMC Palliat Care.

[41] Patel SR, Cole A, Little V (2019). Acceptability, feasibility and outcome of a screening programme for complicated grief in integrated primary and behavioural health care clinics. Fam Pract.

[42] Shear MK, Gribbin Bloom C (2017). Complicated grief treatment: an evidence-based approach to grief therapy. J Ration Emot Cogn Behav Ther.

[43] National Institute for Health and Care Excellence (NICE) (2004). Improving supportive and palliative care for adults with cancer. https://www.nice.org.uk/guidance/csg4/resources/improving-supportive-and-palliative-care-for-adults-with-cancer-pdf-773375005.

